# Clinical characteristics and risk factors of late-stage lung adenocarcinoma patients with bacterial pulmonary infection and its relationship with cellular immune function

**DOI:** 10.3389/fimmu.2025.1559211

**Published:** 2025-04-16

**Authors:** Kangli Yang, Haiting Wei, Weiwei Zhu, Yapeng Xu, Shuaifeng Wang, Feifei Fan, Kai Zhang, Qing Yuan, Hongmin Wang

**Affiliations:** ^1^ Department of Respiratory, The First Affiliated Hospital of Zhengzhou University, Zhengzhou, China; ^2^ College of Public Health of Zhengzhou University, Zhengzhou, China; ^3^ Gene Hospital of Henan Province, The First Affiliated Hospital of Zhengzhou University, Zhengzhou, China; ^4^ Department of Infectious Diseases, The First Affiliated Hospital of Zhengzhou University, Zhengzhou, China

**Keywords:** lung adenocarcinoma, bacterial pulmonary infection, immune function, microbial infection, immune checkpoint inhibitors

## Abstract

**Background:**

To research the clinical characteristics, risk factors, the correlation between bacterial pulmonary infection and immune function of advanced lung adenocarcinoma patients complicated with bacterial pulmonary infection.

**Methods:**

334 stage III and IV lung adenocarcinoma patients admitted to the first affiliated hospital of Zhengzhou University from January 2020 to March 2023 were selected and divided into an infection group (n = 240) and a control group (n= 72) according to whether complicated with bacterial pulmonary infection. The clinical characteristics were analyzed. The pulmonary microbiota and human T lymphocyte subsets (CD3+, CD4+, CD8+) were detected. Multivariate logistic regression analysis was performed to explore the risk factors for pulmonary bacterial infection in advanced lung adenocarcinoma patients.

**Results:**

Among 334 patients, 264 cases were complicated with pulmonary bacterial infection, and 70 cases had no pulmonary bacterial infection. In total, 544 pathogenic bacteria were isolated from the patients. Of these, 170 strains (31.25%) were Gram-negative bacilli, 162 strains (29.78%) were Gram-positive cocci, 27 strains (4.96%) Gram-positive bacilli. There were statistically significant differences in age, smoking, combined diseases, TNM staging, CD3+ T cell percentage, and CD4+ T cell percentage between the two groups (*P* < 0.05). Multivariate logistic regression analysis revealed smoking, bronchiectasis, and diabetes were independent risk factors leading to late-stage lung adenocarcinoma patients with bacterial pulmonary infection (*P* < 0.05). In those patients on immune checkpoint inhibitors, the lung Gram-positive group has a higher number of CD4+ T cells and CD4+/CD8+ T cell ratio than the Gram-negative group (*P* < 0.05).

**Conclusion:**

Smoking, bronchiectasis, and diabetes are risk factors for lung bacterial infection in patients with advanced lung adenocarcinoma. The effect of immune checkpoint inhibitor treatment on T cells is more pronounced in Gram positive bacteria.

## 1 Introduction

Lung adenocarcinoma (LUAD) is a major subtype of lung cancer and the most common form of non-small cell lung cancer (NSCLC) (1, 2). LUAD remains a leading cause of mortality worldwide, with its development intricately linked to disruptions in immune regulation (3, 4). Emerging research highlights the critical role of the lung microbiota, particularly bacterial communities, in lung cancer pathogenesis (5–7). Under normal conditions, the lung microbiota helps maintain immune homeostasis (8–10). However, dysbiosis, or the imbalance of specific bacterial populations, can trigger inflammation, facilitate immune evasion, and promote tumor progression (11, 12). For instance, *veillonella parvula* has been identified as a dominant taxon in dysbiotic profiles that correlate with poor prognosis and increased tumor progression in lung cancer patients (13). Certain bacterial pathogens contribute to an inflammatory microenvironment by secreting metabolites or interacting with host cells, creating conditions conducive to tumor development (14, 15). Therefore, investigating the interplay between lung microbiota dysbiosis, immune modulation, and lung cancer progression offers promising avenues for precision therapy.

Bacterial pulmonary infection (BLI) can influence the development of LUAD (16). BLIs, caused by microbial infections in the lungs, can disrupt the local microbiome and provoke inflammation (17–19). There are increasing reports of pathological and clinical associations between bacterial pneumonia and lung cancer (20, 21). For example, lung cancer patients are particularly susceptible to bacterial pneumonia due to factors such as compromised immune function and airway obstruction (22, 23). Conversely, bacterial infections can exacerbate lung cancer development by inducing chronic inflammation, altering the tumor microenvironment, and enabling immune escape (24–26). Recent studies underscore that bacterial infections may serve as a catalyst for tumor growth (27, 28). However, the pathogenesis of bacterial infection in lung adenocarcinoma patients and how bacteria affect the progression of lung cancer by changing immunity remain poorly understood. Patients with advanced lung adenocarcinoma often have concurrent infection (29, 30). Therefore, we statistically analyzed the clinical characteristics of patients with advanced lung adenocarcinoma, as well as the risk factors for bacterial infection in the lungs and the changes in T cell immune function in these patients, aiming to provide clinical evidence for the selection of appropriate treatment methods.

## 2 Materials and methods

### 2.1 Study subjects and grouping

**Figure 1:**
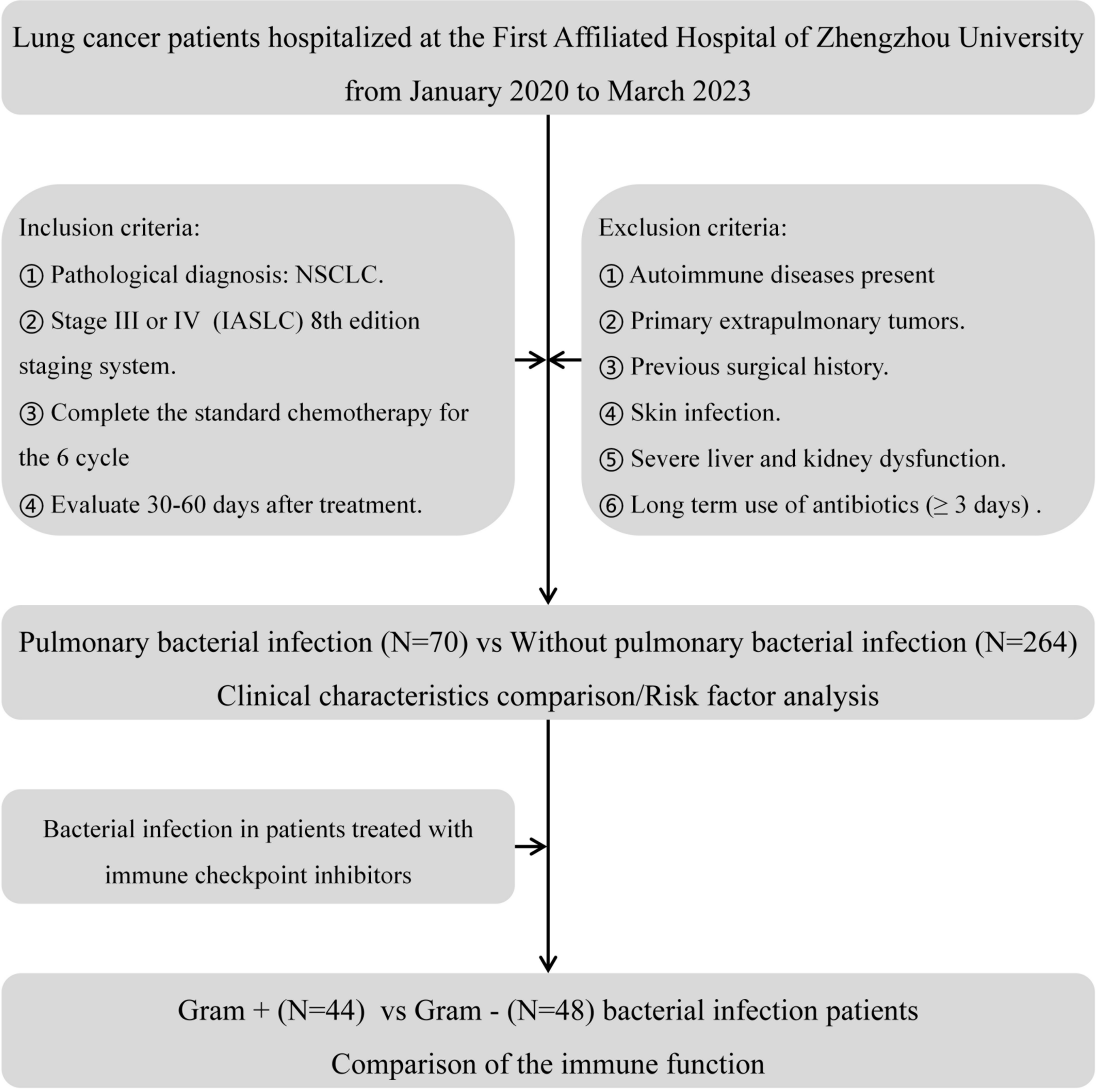
Flowchart of study patients.

This study analyzed the clinical data of patients with advanced lung adenocarcinoma who were admitted to the First Affiliated Hospital of Zhengzhou University between January 2020 and March 2023. The collection and analysis flowchart of patient data is shown in **Figure 1**.

Inclusion criteria: ①Pathologically confirmed diagnosis of NSCLC. ②Disease stage III or IV, classified according to the 8th edition of the International Association for the Study of Lung Cancer (IASLC) staging system. ③Patients who completed the sixth cycle of standard chemotherapy were assessed 30-60 days post-treatment.

Exclusion criteria: ①Presence of autoimmune diseases. ②Diagnosis of primary extrapulmonary tumors. ③History of prior surgical intervention. ④Presence of skin infections, such as diabetic foot ulcers or open wounds. ⑤Severe hepatic or renal dysfunction, or bone marrow suppression leading to irregular chemotherapy cycles. ⑥Prolonged use of antibiotics (≥3 days) before blood collection.

Patients were categorized into two groups based on the presence of bacterial pulmonary infections, determined through sputum smear results, bronchoalveolar lavage fluid Next Generation Sequencing (NGS), or chest high-resolution computed tomography (HRCT). The two cohorts included those with late-stage LUAD and BLI (LUAD-BLI group) and those with late-stage LUAD without BLI (LUAD group).

This study was approved by the Ethics Committee of the First Affiliated Hospital of Zhengzhou University (approval number: 2022-KY-0677-003, Supplementary Materials 1).

### 2.2 DNA extraction, library construction, and high-throughput sequencing

The microbial genomic DNA (gDNA) was extracted using the Zymo Research BIOMICS DNA Microprep Kit (Product Code: D4301). The quality of the extracted gDNA was assessed through 0.8% agarose gel electrophoresis, and its concentration was measured using the Tecan F200 system with the PicoGreen dye-based quantification method. Specific primers targeting the full-length 16S rRNA gene, including the forward primer 8F (5’AGAGTTTGATCATGGCTCAG3’) and the reverse primer 1492R (5’CGGTTACCTTGTTACGACTT3’), were employed to amplify the target region of the samples. Each sample was processed in triplicate, and the PCR reactions were stopped during the linear amplification phase. The PCR amplicons from the same sample were pooled and analyzed by electrophoresis. Subsequently, the desired DNA fragments were excised from the gel and purified using a gel extraction kit, followed by washing and elution with TE buffer. The purified PCR products were quantified using the Qubit 2.0 Fluorometer. After quality control verification, the Nanopore R9.4.1 library preparation kit was utilized to construct sequencing libraries. Finally, the libraries were sequenced on the MinION platform, and real-time high-accuracy base calling was performed during the sequencing process.

### 2.3 Baseline indicators

Medical records were reviewed to collect crucial clinical characteristics.

General information: gender, age, tumor node metastasis (TNM) staging, Performance Status (PS score), immune checkpoint inhibitor treatment.

Clinical characteristics: underlying diseases, smoking history, body mass index (BMI).

Laboratory indicators: CD3+ T cell percentage, CD4+ T cell count, CD8+T cell count, CD4+/CD8+T cell ratio, sputum smear or bronchoalveolar lavage fluid NGS test results.

Survival follow-up: Follow up will be conducted after 1 year, and if the patient dies during this period, it will be marked as poor prognosis.

### 2.4 Statistical analysis

All data were analyzed using SPSS 21.0 software. Continuous variables are expressed as mean ± standard deviation and compared between groups through student *t*-test. When the sample size within the group is less than 30 and does not follow a normal distribution, rank sum test is used for interring group comparison. Categorical variables are expressed as percentages (%) and chi-square tests are used for interring group comparisons. After single factor screening, multiple logistic regression analysis was used to determine the relevant independent risk factors, and all relevant hazard ratios (HR) and 95% confidence intervals (CI) were calculated. *P* < 0.05 is considered statistically significant.

## 3 Results

### 3.1 Detection of pathogens in patients with lung adenocarcinoma complicated by pulmonary infection

A total of 544 strains of pathogenic bacteria were isolated from the infected individuals, among which 162 strains of Gram-positive cocci were detected, accounting for 29.78%; 27 strains of Gram-positive bacteria were detected, accounting for 4.96%; 170 strains of Gram-negative bacteria were detected, accounting for 31.25%; 38 strains of Candida were detected, accounting for 6.99%, 38 strains of aspergillus were detected, accounting for 6.99%. In addition, 109 strains of non-bacterial infections, such as SARS-CoV-2, Influenza, Epstein-Barr virus, Cytomegalovirus, mycobacterium tuberculosis, nontuberculous mycobacteria, Mycoplasma, non-bacterial infections were also detected, accounting for 20.0% of the total (**Table 1**).

**Table 1:** Detection of pathogens in patients with adenocarcinoma of the lung complicated with pulmonary infection.

Pathogen	Number of Strains (n/strain)	Proportion (%)
Gram-positive cocci		
Streptococcus pneumoniae0	152	27.94
*Staphylococcus aureus*	4	0.74
Enterococcus faecalis	3	0.55
Enterococcus cloacae	3	0.55
Total	162	29.78
Gram-positive bacilli	27	4.96
Gram-negative bacilli		
Escherichia coli	91	16.73
Haemophiles influenzae	28	5.15
Klebsiella pneumoniae	10	1.84
Pseudomonas aeruginosa	30	5.51
Acinetobacter baumannii	11	2.02
Total	170	31.25
Candida	38	6.99
Aspergillus	38	6.99
SARS-CoV-2	14	2.57
Influenza	17	3.13
Epstein-Barr virus	25	4.60
Cytomegalovirus	23	4.23
Mycobacterium tuberculosis	6	1.10
Nontuberculous mycobacteria	3	0.55
Mycoplasma	21	3.86

### 3.2 Baseline clinical data

The 334 advanced stage lung adenocarcinoma patients were then divided into a group of lung cancer patients with bacterial infections (LUAD-BLI group, n = 264) and a group of lung cancer patients without bacterial infection (LUAD group, n = 70) based on whether there is a bacterial infection in the lungs. We examined the baseline clinical characteristics of lung adenocarcinoma patients and observed some significant difference. Compared with the non-infected group, the infected group is older in age. Compared to non-smokers, the number of infected patients in the smoking population has significantly increased. The infection rate of stage 4 patients has increased compared to stage 3 patients. The infection rate in patients with bronchiectasis and diabetes was significantly higher. Compared with the non-infected group, the proportion of CD3+ T cell percentage and the CD4+ T cell count in the infected group patients was significantly reduced. In addition, there were no statistically significant differences in other clinical indicators such as gender, BMI, chronic obstructive pulmonary disease, hypertension, PS score, CD8+T cell count and CD4+/CD8+T cell ratio between the two groups (**Table 2**).

**Table 2:** Baseline characteristics of lung cancer patients according to bacterial infection.

Variable	Total (n=334)	LUAD-BLI group (n=264)	LUAD group (n=70)	X^2^/t	*P*
Age (years)	58.5 ± 11.3	59.31 ± 10.79	55.21 ± 12.63	2.718	0.007
Sex, n (%)					
Male	185 (55.4)	151 (81.6)	34 (18.4)	1.666	0.197
Female	149 (44.6)	113 (75.8)	36 (24.2)		
Smoking, n (%)					
Never smoking	234 (70.1)	175 (74.8)	59 (25.2)	8.544	0.001
smoking	100 (29.9)	89 (89.0)	11 (11.0)		
Body Mass Index, n (%)					
<18.5kg/m²	53 (15.8)	46 (86.8)	7 (13.2)	4.430	0.109
18.5-24kg/m²	249 (74.6)	190 (76.3)	59 (23.7)		
>24kg/m²	32 (9.6)	28 (87.5)	4 (12.5)		
Stage, n (%)					
III	276 (82.7)	212 (76.8)	64 (23.2)	4.773	0.029
IV	58 (17.3)	52 (89.7)	6 (10.3)		
Chronic obstructive pulmonary disease					
No	254 (76.0)	195 (76.8)	59 (23.2)	3.299	0.069
Yes	80 (24.0)	69 (86.2)	11 (13.8)		
Bronchiectasis					
No	268 (80.2)	203 (75.7)	65 (24.3)	8.892	0.003
Yes	66 (19.8)	61 (92.4)	5 (0.76)		
Diabetes					
No	260 (77.8)	193 (74.2)	67 (25.8)	16.397	0.000
Yes	74 (22.2)	71 (95.9)	3 (4.1)		
hypertension					
No	281 (84.1)	224 (79.7)	57 (20.3)	0.485	0.486
Yes	53 (15.9)	40 (75.5)	13 (24.5)		
Performance Status, n (%)					
0	69 (20.6)	51 (73.9)	18 (26.1)	7.539	0.110
1	122 (36.7)	92 (75.4)	30 (24.6)		
2	100 (29.9)	81 (81.0)	19 (19.0)		
3	38 (11.3)	35 (92.1)	3 (7.9)		
4	5 (1.5)	5 (100)	0 (0)		
T cell subpopulation					
CD3+ T cell percentage, n (%)	64.4 ± 11.2	63.45 ± 11.34	67.78 ± 10.23	2.895	0.004
CD4+ T cell count	538.2 ± 320.5	511.17 ± 314.55	640.20 ± 324.65	3.031	0.003
CD8+ T cell count	421.3 ± 365.4	402.22 ± 386.27	493.25 ± 262.50	1.860	0.064
CD4+/CD8+ T cell ratio	1.5 ± 0.9	1.47 ± 0.94	1.47 ± 0.71	0.053	0.958

### 3.3 Logistic regression analysis of factors influencing the advanced stage lung adenocarcinoma patients with concurrent infections

In the univariate analyses (**Table 3**), infection was significantly associated with age (OR: 1.032, 95% CI: 1.008-1.056; *P* = 0.008), smoking (OR: 2.728, 95% CI: 1.365-5.452; *P* = 0.005), bronchiectasis (OR: 3.906, 95% CI: 1.505-10.137; *P* = 0.005), Diabetes (OR: 8.216, 95% CI: 2.504-26.958; *P* = 0.001), stages (OR: 2.616, 95% CI: 1.074-6.372; *P* = 0.034), CD3+T cell percentage (OR: 0.966, 95% CI: 0.943-0.989; *P* = 0.005), and CD4+T cell count (OR: 0.999, 95% CI: 0.998-1.000; *P* = 0.004). In the multivariate analyses, infection was independently associated with smoking (OR: 2.305, 95% CI: 1.091-4.867; *P* = 0.029), bronchiectasis (OR: 5.054, 95% CI: 1.885-13.554; *P* = 0.001), and Diabetes (OR: 7.290, 95% CI: 2.146-24.760; *P* = 0.001).

**Table 3:** Cox regression analysis of variables impacting advanced stage lung adenocarcinoma patients with concurrent infections.

Variable	Univariate			Multivariate		
	OR (95% CI)	*P*	*P.*adjust	OR (95% CI)	*P*	*P.*adjust
age	1.032 (1.008~1.056)	0.008	0.01			
smoking	2.728 (1.365~5.452)	0.005	0.007	2.305 (1.091~4.867)	0.029	0.032
bronchiectasis	3.906 (1.505~10.137)	0.005	0.007	5.054 (1.885~13.554)	0.001	0.003
Diabetes	8.216 (2.504~26.958)	0.001	0.003	7.290 (2.146~24.760)	0.001	0.003
stages	2.616 (1.074~6.372)	0.034	0.034			
CD3+T cell percentage	0.966 (0.943~0.989)	0.005	0.007			
CD4+T cell count	0.999 (0.998~1.000)	0.004	0.007			

### 3.4 In patients treated with immune checkpoint inhibitors, the number of CD4+T cell and the ratio of CD4+/CD8+ T cell in the group with Gram-positive bacteria in the lungs are higher than those in the group infected with Gram-negative bacteria

Immune checkpoint inhibitors are widely used in the treatment of advanced lung adenocarcinoma. After their application, the number of cytotoxic T cells in tumor tissues often increases. On the other hand, the above results show that if bacterial infection occurs in the lungs, the number of T cells in patients will decrease. In view of this, it is of great significance to explore the impact of immune checkpoint inhibitors on cellular immunity in patients for optimizing treatment regimens. Different bacteria lead to different changes in cellular immunity. By distinguishing these changes, the role of bacteria in immune checkpoint treatment can be clarified. Therefore, we divided lung adenocarcinoma patients treated with immune checkpoint inhibitors and complicated with pulmonary bacterial pneumonia infection into two groups according to the types of bacteria: the group with immune checkpoint treatment combined with Gram-negative bacteria and the group with immune checkpoint treatment combined with Gram-positive bacteria. The results showed that compared with the group with Gram-negative bacteria, the number of CD4+ T cells and the ratio of CD4/CD8 in the immune inhibitor treatment group of the Gram-positive bacteria group were higher (**Table 4**).

**Table 4:** Differences in T cell subpopulation in immune checkpoint inhibitor treatment for lung adenocarcinoma patients with pulmonary bacterial pneumonia infection by Gram-positive (G-) vs Gram-negative (G+) bacteria.

T cell subpopulation	G- infection with immune checkpoint inhibitors(n=48)	G+ infection with immune checkpoint inhibitors(n=44)	*P*	*P.adjust*
CD3+ T cell percentage	65.24 ± 12.90	66.29 ± 10.10	0.6697	0.8654
CD4+ T cell count	500.6 ± 266.9	627.0 ± 284.3	0.0314	0.0408
CD8+ T cell count	395.5 ± 170.2	386.0 ± 156.5	0.7835	0.8126
CD4+/CD8+ T cell ratio	1.377 ± 0.681	1.826 ± 1.153	0.0252	0.0391

## 4 Discussion

The occurrence and development of LUAD are complex, multi-step process involving the interaction of genetic and environmental factors (31–33). In recent years, the relationship between lung microbiota, including bacteria, viruses, and fungi, and lung cancer has garnered increasing attention (5, 34, 35). The lung microbiota, which is composed of bacteria, fungi, and viruses, plays a critical role in respiratory health. It interacts with host immunity and maintains microbial balance (36). Dysbiosis disrupts this equilibrium. It contributes to chronic inflammation, fibrosis, and malignancy through pathogen overgrowth, toxin production, and aberrant immune signaling (37). Research indicates that lung microbiota can influence the development of cancer by regulating the host’s immune system (38, 39). Additionally, lung cancer patients often suffer from pulmonary bacterial infections. Alterations in the pulmonary bacterial community may impact immune surveillance, thereby affecting the growth and progression of lung cancer (29, 40). Consequently, understanding the clinical characteristics of bacterial lung infections in lung cancer patients and their relationship with cellular immunity offers valuable insights into how microbial communities affect the onset and progression of lung cancer through immune mechanisms.

In this study, the infection rate of Gram-negative bacteria was the highest, at 31.25% (170/544). Compared to other bacteria, Gram-negative bacteria dominated, which was consistent with the following research findings (16, 41). The first reason is the outer membrane of Gram-negative bacteria contains lipopolysaccharide (LPS). LPS not only protects bacteria from environmental stress, such as antibiotics and immune system attacks, but also has a strong immunostimulatory effect. This effect triggers a more severe inflammatory response (42, 43). Moreover, the pore proteins in the outer membrane have low selectivity and are difficult for antibiotics to penetrate. This makes Gram-negative bacteria more resistant to antibiotic treatment (44, 45). Among Gram-negative bacteria, *Escherichia coli* accounted for 91 cases, representing 16.73% (91/544), followed by *Pseudomonas aeruginosa* at 5.51% (30/544). Studies have shown that persistent infections by Gram-negative bacteria, such as *Pseudomonas aeruginosa* or *Klebsiella* pneumoniae, can lead to chronic inflammation. This inflammation produces a microenvironment. The microenvironment is conducive to cancer development as it promotes cell proliferation, angiogenesis, and immune evasion (46, 47). Therefore, the preventing and controlling Gram-negative bacterial infections and the managing antibiotic resistance are key focuses in the treatment of pulmonary infections.

Considering the high incidence rate of lung cancer-associated infection and its impact on patients’ survival rate, understanding the predictive factors of the development of lung cancer-associated infection is the first step for clinicians to establish a monitoring program. This investigation analyzed the risk factors for bacterial lung infection in patients with advanced lung adenocarcinoma and found that age, smoking, stage, bronchiectasis, diabetes, CD3+ T cell percentage, and CD4+ T cell count were closely related to bacterial lung infection. Smoking, bronchiectasis, and diabetes were determined to be predictive factors of bacterial infection in the lungs of patients with advanced lung adenocarcinoma. Advanced age has been shown to increase the risk of bacterial lung infection in patients with lung adenocarcinoma. These patients have weakened lung function, decreased resistance, and are thus more susceptible to bacterial invasion. The later the stage of lung cancer, the higher the risk and severity of bacterial lung infection. This association is caused by the impact of the tumor itself on the airway and immune system and the side effects of treatment measures, such as chemotherapy and radiotherapy (48, 49). Strengthening infection management and providing comprehensive treatment for lung cancer patients are important parts of improving survival rate and quality of life (50). Moreover, there is a close two-way interaction between bronchiectasis and lung infection in lung cancer patients. Bronchiectasis provides a good breeding ground for pathogens, increasing the risk of infection and the difficulty of treatment in lung cancer patients. Meanwhile, lung cancer and its treatment may aggravate the condition of bronchiectasis, forming a vicious cycle (51, 52). Effective airway management, infection control, and comprehensive treatment are the keys to improving patient prognosis. Furthermore, diabetes and lung cancer work together to significantly increase the incidence and complexity of lung infections. Diabetes greatly increases the susceptibility of lung cancer patients to infection through immune function suppression, a hyperglycemic environment, and metabolic disorders. Lung cancer and its treatment further increase the risk of infection (42). Therefore, preventing and controlling of pulmonary infection in patients with diabetes and lung cancer requires focusing on glycemic control, individualized anti-infective treatment and nutritional support. This is of great significance for improving the patient’s prognosis.

While both B and T lymphocytes play critical roles in the immune response against tumors, it is the T lymphocytes that are particularly effective in mediating cellular immunity against tumors (53, 54). T lymphocytes, the primary contributors, are essential for recognizing and targeting tumor cells. They help to prevent the growth, and spread of cancer (55). T lymphocytes are further classified into different types based on their functions. These types include delayed hypersensitivity T cells, killer T cells, cytotoxic T cells, and helper T cells. Each type of T lymphocyte plays a distinct role in the immune system’s defense mechanisms (56). Among these, CD4+ T cells, which are markers of helper T cells, are crucial for anti-tumor immunity. CD4+ T cells assist other immune cells in recognizing and responding to cancer cells. As a result, they amplify the immune response (57). On the other hand, CD8+ T cells include both cytotoxic T cells and suppressor T cells. Cytotoxic T cells are directly involved in killing tumor cells, while suppressor T cells can regulate immune responses by dampening excessive immune activity (58). In our results, the proportions of CD4+ cells and CD8+ cells were decreased. This suggests that bacterial infection can affect the immune function of patients with lung adenocarcinoma. Moreover, while both CD4+ and CD8+ T cells contribute to anti-tumor immunity, the balance between these two cell types is crucial for maintaining effective immune function. The results of the study showed that the ratio of CD4+ to CD8+ cells has decreased. In this study, we conducted a multivariate logistic regression analysis to explore the relationship between T lymphocytes and infection. The results revealed that T lymphocytes were not an independent factor influencing infection. This finding suggests that their role in immune defense may be more complex than previously thought. It implies that lung immune dysfunction does not directly lead to bacterial infections. Instead, it may be influenced by a combination of other factors. One such factor is imbalances in the respiratory flora, which could contribute to infection susceptibility (59). Thus, the interaction between immune dysfunction and environmental factors, such as the microbiome, may play a significant role in determining the overall immune response to infections and cancer.

Studies have shown that bacteria and their metabolites can directly act on T cells, interfere with their normal metabolism, and inhibit their proliferation (60). Bacterial pathogens use metabolic and molecular strategies to evade immune responses, including cytokine manipulation (e.g., Mycobacterium tuberculosis and Bordetella pertussis), metabolic interference (e.g., via short-chain fatty acids and pathogens like Salmonella), and commensal microbiota interactions (gut microbiota engaging with pattern recognition receptors) (10, 61, 62). ICIs, which block T-cell inhibitory receptors, have dual effects. They increase susceptibility to certain infections, such as disrupting *M. tuberculosis* control and triggering T-cell responses against skin microbiota (63). Immunosuppressive therapies for ICI-related side effects also heighten infection risks (64). Patients with advanced lung cancer often use immunosuppressant therapy and are prone to bacterial infections (63, 65). Therefore, analyzing different types of bacterial infections can provide guidance for adjusting immunosuppressant therapy. We found during data analysis that Gram-positive and Gram-negative bacteria may have different effects on the immune system of cancer patients. In lung adenocarcinoma patients treated with immune checkpoint inhibitors, those with Gram-positive bacterial pneumonia show higher CD4+ T cell counts and CD4+/CD8+ T ratios than those with Gram-negative infections. This finding underscores the need to study how these inhibitors affect cellular immunity and the role of bacteria. Many reports have paid attention to infection risk and prognosis caused by ICIs use (66, 67). In recent years, some research has still focused on how bacterial infections directly impact the efficacy of immune checkpoint inhibitors and the modulation of T cells. For example, mice were engrafted with MC38 colon adenocarcinoma or B16-OVA melanoma cells, the tumor volumes of non-*H.pylori*-infected mice undergoing anti-CTLA-4 and/or PD-1 or anti-cancer vaccine treatments were significantly smaller than those of *H.pylori*-infected mice. They observed a decreased number and activation status of tumor-specific CD8+ T cells in the tumors of infected mice treated with cancer immunotherapies independent of the gut microbiome composition (68). In more in-depth mechanism research, the immune checkpoint T-cell immunoglobulin and ITIM domain (TIGIT) was significantly upregulated on lymphocytes of S. Typhimurium-infected C57BL/6 mice, particularly on CD4+ T cells. This drastically limited their proinflammatory function (69). These studies and our results indicate that different bacterial infections can affect the use of immunosuppressants, but it is very difficult to explain the mechanism behind this phenomenon. Our findings suggest that immune checkpoint inhibitors may have better therapeutic effects in patients with Gram-positive infections, which can provide a reference for treatment strategies. Future research should focus on the specific impacts of ICIs on T cell subsets during Gram-positive and Gram-negative infections to clarify their roles. Despite the clinical success of immune checkpoint inhibitors, a significant subset of patients experiences immune-related adverse effects (irAEs) that can limit treatment efficacy and tolerability. Factors influencing ICI response and mechanisms of resistance, including immune dysregulation and host-specific variables, have been extensively reviewed (70). Understanding these mechanisms is crucial for optimizing patient selection and improving therapeutic strategies.

## 5 Conclusion

In conclusion, age, diabetes, and bronchiectasis are independent risk factors for pulmonary bacterial infections in patients with advanced lung adenocarcinoma. Understanding these risk factors is crucial for effective prevention, early diagnosis, and timely treatment in clinical practice. The effect of immune checkpoint inhibitor treatment on T cells is more pronounced in Gram positive bacteria. However, the sample size in this study is quite small, and no long-term follow-up was conducted. As a result, there may be potential bias in the findings. To confirm these results, further research involving a larger sample size and long-term follow-up through a multicenter study is needed.

## Data Availability

The original contributions presented in the study are included in the article/**Supplementary Material**. Further inquiries can be directed to the corresponding author.
