# Supplementary figures and images for: Clinical characteristics and risk factors of late-stage lung adenocarcinoma patients with bacterial pulmonary infection and its relationship with cellular immune function
Source: Front Immunol. 2025 Apr 16;16:1559211. doi: 10.3389/fimmu.2025.1559211 (PMC12040822; doi:10.3389/fimmu.2025.1559211)

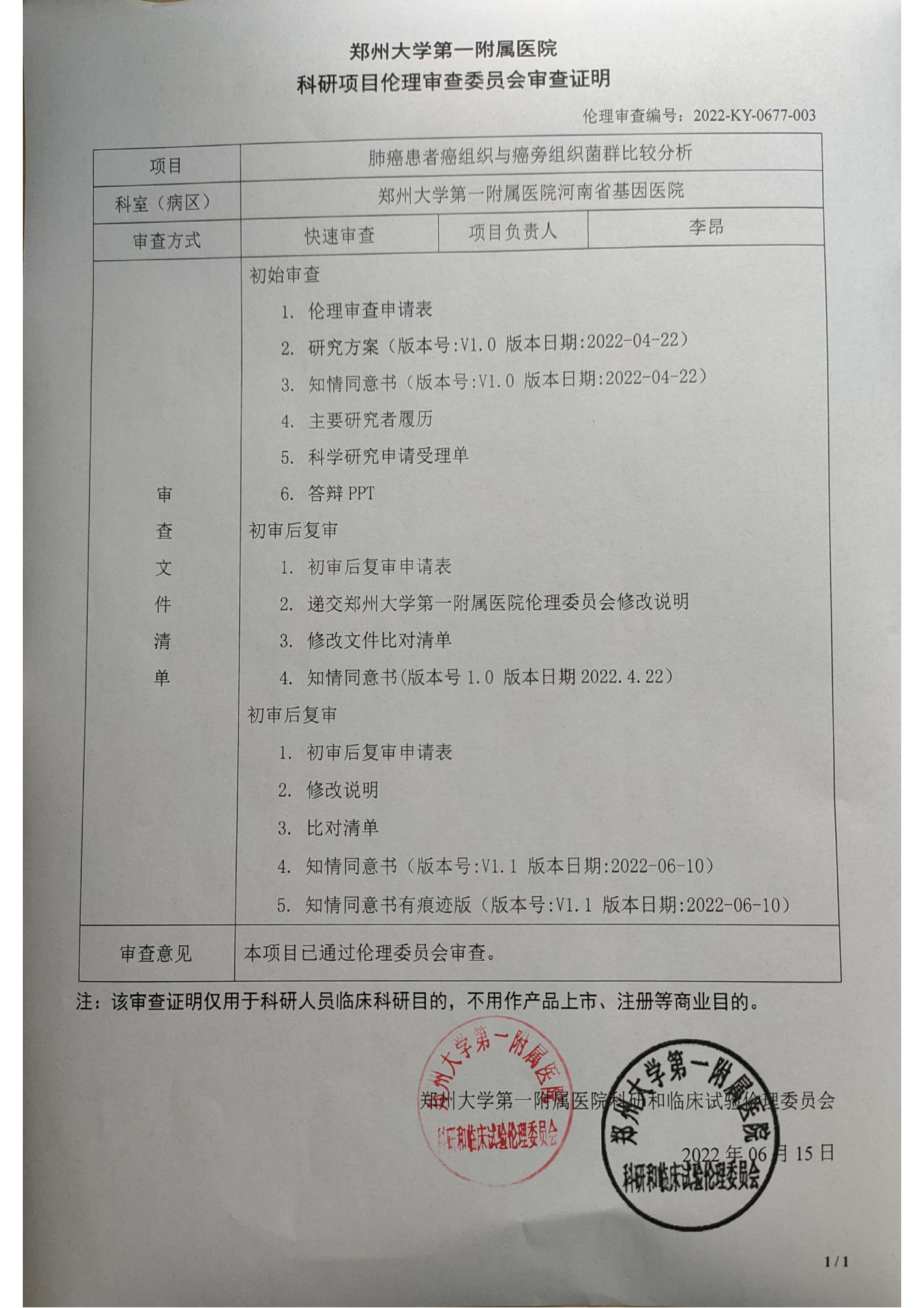

Supplement: Supplementary file 1 [file Image1.png]
